# Research on Construction Workers’ Activity Recognition Based on Smartphone

**DOI:** 10.3390/s18082667

**Published:** 2018-08-14

**Authors:** Mingyuan Zhang, Shuo Chen, Xuefeng Zhao, Zhen Yang

**Affiliations:** 1 Department of Construction Management, Dalian University of Technology, Dalian 116000, China; chen_shuoa@163.com (S.C.); yangz@mail.dlut.edu.cn (Z.Y.); 2 School of Civil Engineering, Dalian University of Technology, Dalian 116000, China; zhaoxf@dlut.edu.cn

**Keywords:** sensor, smartphone, feature extraction, machine learning, activity recognition, construction management

## Abstract

This research on identification and classification of construction workers’ activity contributes to the monitoring and management of individuals. Since a single sensor cannot meet management requirements of a complex construction environment, and integrated multiple sensors usually lack systemic flexibility and stability, this paper proposes an approach to construction-activity recognition based on smartphones. The accelerometers and gyroscopes embedded in smartphones were utilized to collect three-axis acceleration and angle data of eight main activities with relatively high frequency in simulated floor-reinforcing steel work. Data acquisition from multiple body parts enhanced the dimensionality of activity features to better distinguish between different activities. The CART algorithm of a decision tree was adopted to build a classification training model whose effectiveness was evaluated and verified through cross-validation. The results showed that the accuracy of classification for overall samples was up to 89.85% and the accuracy of prediction was 94.91%. The feasibility of using smartphones as data-acquisition tools in construction management was verified. Moreover, it was proved that the combination of a decision-tree algorithm with smartphones could achieve complex activity classification and identification.

## 1. Introduction

Analyzing and tracking workers’ activity in a timely and effective manner is significant in workers’ production-efficiency evaluation and schedule monitoring [1]. In building manufacturing processes, the low efficiency of construction workers inevitably leads to low productivity, resulting in the waste of time and resources and economic losses for whole projects. The first step to solve the problem is to accurately monitor and evaluate labor consumption. The results are compared with the project baseline to address relevant problems [2].

The traditional monitoring approach of direct observation wastes human resources and is vulnerable to the subjectivity of researchers [3]. Automated data acquisition has a clear advantage in tracking and monitoring labor. The initial research in classification and identification of workers’ activities was mainly based on vision-based sensing technologies. Favela et al. [4] extracted body postures from videos recorded by wireless cameras to identify workers’ activities. However, although the massive image data was conducive to improving recognition accuracy, it required more resources at a higher cost. To make activity recognition more efficient and intelligent, wearable inertial measurement units (IMUs) composed of accelerometers and gyroscopes have been utilized by computer-science researchers to classify and identify different human activities since 2000. This kind of technique improved the efficiency and quality of studies, but wearing external sensors will limit the flexibility of sensors and add hardware costs, limiting their application on construction sites. To remedy this situation, researchers have shifted their focus since 2008 to smartphones in the field of data acquisition. The smartphone is not only an intelligent tool for communication, socializing, shopping, transportation, and entertainment in modern life, it integrates a sensor-based platform that combines sensors, such as accelerometers, gyroscopes, magnetic sensors, pressure sensors, and temperature sensors. Smartphones are used in the field of human-activity recognition as data-acquisition tools based on multiple embedded sensors. For example, accelerometers have been embedded in smartphones and used to collect data [5,6,7], and GPS devices have also been used [8,9,10], as well as gyroscopes [11] and pressure sensors [12]. The smartphone can become an information-management platform by virtue of its data-processing ability and overall performance. Also desirable is that a smartphone is an open-source system with a modern software-development environment, which is beneficial in information collection and data processing [13]. Emiliano et al. built a human-activity recognition system based on a Nokia N95 smartphone [14]. However, only a few types of pre-set activities were selected, such as walking, standing, sitting, and running. After 2010, researchers began to expand their research areas in human activity recognition based on smartphones, adding more complex daily activities such as driving, cycling, riding an elevator, and jumping to broaden the study field [12,15,16]. Liju and Koshy [17] utilized video annotation and a decision tree algorithm to determine the placement of sensors for masonry workers’ activity recognition. The results showed that the placement of sensors has a significant impact on activity-recognition accuracy. Figo and Diniz prepared a summary analysis of sensing data preprocessing techniques and feature extraction [18]. The selection of features was analyzed quantitatively. Additionally, the recognition accuracy for different combinations of features was verified through experiments. A comparative analysis of classification algorithms was conducted in the area of human-activity recognition [3], and these were subsequently perfected. The combination of various algorithms was proved to promote the accuracy of human-activity identification. Literature on human-activity recognition based on smartphones was summarized, with an overview of action types, system platforms, and feature extraction and classification algorithms [19,20]. However, these studies of human activity and status are widely applied to the fields of health, exercise, and medical care, and there has been little research in construction management. In addition to convenient data acquisition, the embedded CPU, GPU, memory cards, wireless adapters, and operating systems, i.e., Android and iOS, make the smartphone capable of data storage and wireless data transmission and processing. These attributes give the smartphone the potential to be an interactive platform enabling intelligent management in a complex construction environment [13]. Despite the smartphone having great potential for construction automation, it has not been fully explored in the field of engineering management.

Little research has been conducted on workers’ activity management in the field of construction management, and the work has been at the primary stage. Most initial research was conducted through visual technology. With the development and application of sensor-based technology, some researchers have explored these means to implement the identification management of construction workers’ activities. However, the few types of construction activities in previous studies were of a simple nature. Consequently, this paper proposes an activity-recognition method for construction workers based on the smartphone.

**Figure 1:**
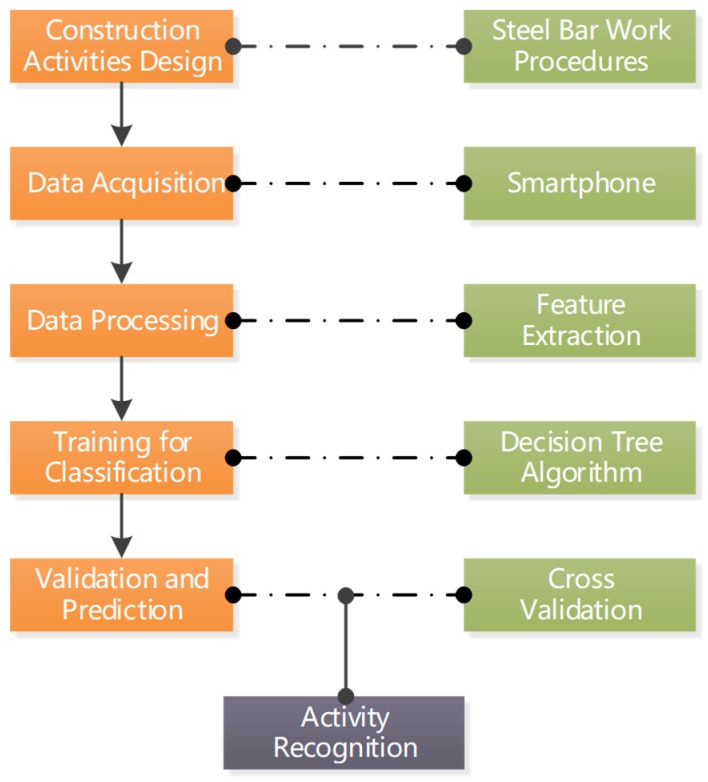
The framework of construction workers’ activity.

The productivity level in the construction industry is often determined by the construction phase, which consumes longer time periods throughout a project lifecycle. In addition, the construction of the main structure plays a leading role in the entire project. Therefore, strengthening management and supervision of the construction process will improve overall productivity. Many construction technologies in concrete work, such as concrete mixing, transportation, and pouring, adopt mechanized operations, which greatly reduces the labor and time cost. Compared with the continuous and mechanized operations in concrete work, there are still many manual operations with a high degree of risk and uncertainty in reinforcing steel work. Therefore, this paper takes construction activities in floor-reinforcing steel work as a research object. The framework of activity recognition was established as shown in Figure 1. Two smartphones were used to collect three-axis acceleration and angle information of eight main activities related to floor-reinforcing steel work. The eight main activities consisting of standing, walking, squatting, cleaning up the template, fetching and placing a rebar, locating the rebar, banding the rebar, and placing concrete pads. Data acquisition from multiple body parts enhanced the dimensionality of motion features, making the distinction between each activity more apparent. Subsequently, the classification and identification of construction workers’ activities was implemented through data-processing technology with data mining, providing a new perspective to promote labor management and improve management efficiency.

## 2. Literature Review

Due to the complexity of construction activities, previous studies for construction activity classification and recognition have been mainly based on image-acquisition technology, with the assistance of sensor-based technologies. Based on the integration of image-acquisition technology and sensor-based location technology, Tao et al. [2] combined workers’ spatial position information with physiological states to quantitatively analyze construction efficiency. Tharindu and Janaka [21] used digital video images, audio files, and thermal images to judge whether the workers were in a working condition. However, image-acquisition technology is vulnerable to the impact of the surrounding environment, such as lighting conditions and background colors, and it demands massive data storage, which limits its application in a construction environment. In this context, the advantage of sensor-based technologies in data collection is becoming obvious. Since 2011, researchers have shifted their focus from image-acquisition technology to sensor-based data-collection technology. Liju and Koshy [22] were among the first researchers who explored the application of accelerometer in construction activity recognition. They used accelerometers to establish a framework for classifying three main activities of masonry work. The classification algorithms were subsequently compared and analyzed. In a subsequent study [23], the activities of carpenters and steel fixers were classified. Nevertheless, the types of preset activities consisted of a few easy tasks. However, one single sensor-based technology cannot meet the management requirements of a complex construction environment. In contrast, integrated multiple sensor-based technologies usually lack systemic flexibility and stability. More recently, with the development and popularization of smartphones, researchers have used smartphone-based data acquisition and activity identification for data-driven simulation of construction activity [24]. As a single-node integrated platform of multiple sensor-based technologies, a smartphone can satisfy the dimensional demands of data acquisition and adapt to a complex construction environment. For example, Akhavian and Behzadanb [3] used wearable IMUs of ubiquitous smartphones to recognize brickwork activities performed by subjects in experiment environment. The smooth transmission of data information was implemented even under a high collection frequency. They also compared the capability of various classification algorithms. A neural network, decision tree, K-nearest neighbor (KNN), signal vector magnitude (SVM), and logistic regression were applied separately to obtain the classification of activities. The results showed that the neural network had the highest recognition accuracy in identifying masonry workers’ activity. However, the selection of smartphone’s position or activity recognition was deficient. The upper arm was not expected to provide accurate signals.

## 3. Research Method

**Figure 2:**
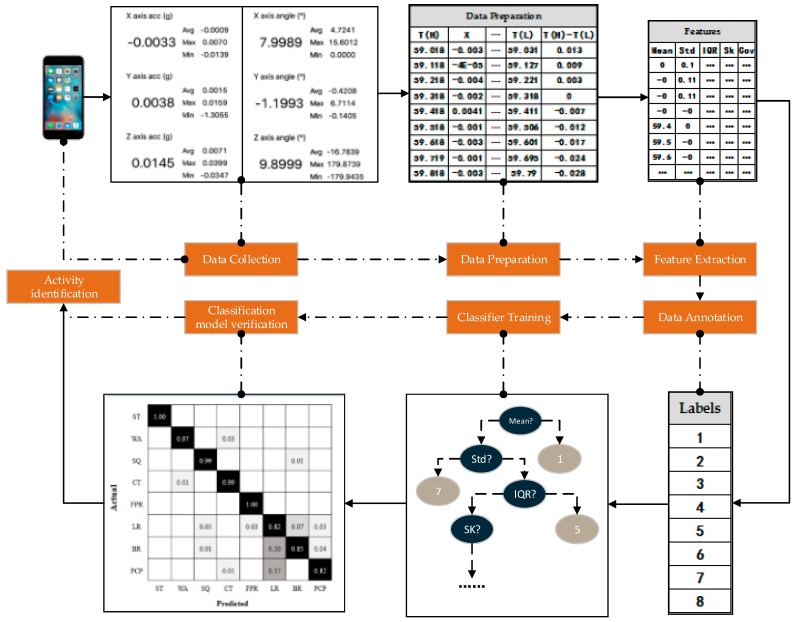
Framework of construction workers’ activity identification based on smartphones.

This study adopted smartphones as data-acquisition tools. A sliding window technique was used for motion classification. The raw data of motions was segmented by an appropriate time window to better identify the properties of each motion in varying states and provide a prerequisite for feature extraction. Subsequently, by labeling extracted features, a motion-classification model was established based on a supervised learning algorithm. After evaluation and verification, the model could classify and identify the different activities of construction workers. The process of activity identification is shown in Figure 2.

### 3.1. Data Collection Using Smartphones

In this research, accelerometer and gyroscope sensors embedded in a smartphone were utilized to collect motion data of construction workers. The accelerometer sensor can measure the acceleration of a device in three axes of X, Y and Z. A gyroscope measures the rotation rate of the device by detecting the roll, pitch, and yaw motions of the smartphone in X, Y and Z axes [3]. When a smartphone is attached to the construction worker participating in different activities, these two sensors can acquire relevant signals. In order to enhance the dimensionality of motion features and make the distinction between each activity more apparent, two smartphones were used to collect data.

**Figure 3:**
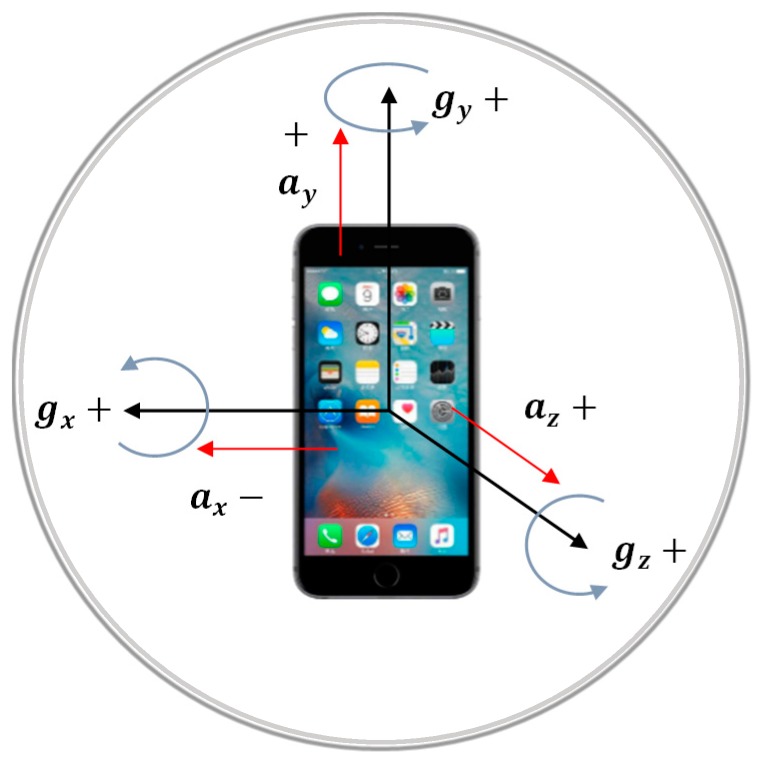
The three-axis coordinate system of built-in sensors.

To ensure the stability and consistency of experimental data, this research adopted two iPhone 6s smartphones with the iOS 10.3.2 operating system as data-acquisition tools. The built-in Invensense_MP67B sensor can collect three-axis acceleration ($a_{x}\;a_{y}\;a_{z}$) and three-axis angles ($g_{x}\;g_{y}\;g_{z}$). The three-axis coordinate system of sensors embedded in the smartphone is shown in Figure 3, where a indicates acceleration and g indicates angle. The Orion-CC app, which can acquire three-axis acceleration and angle data and provide geographic location information, was developed by the Xuefeng Zhao team from Dalian University of Technology. The accuracy and reliability of data acquisition and its environmental adaptation were verified in cable force measurement.

### 3.2. Data Preparation

**Figure 4:**
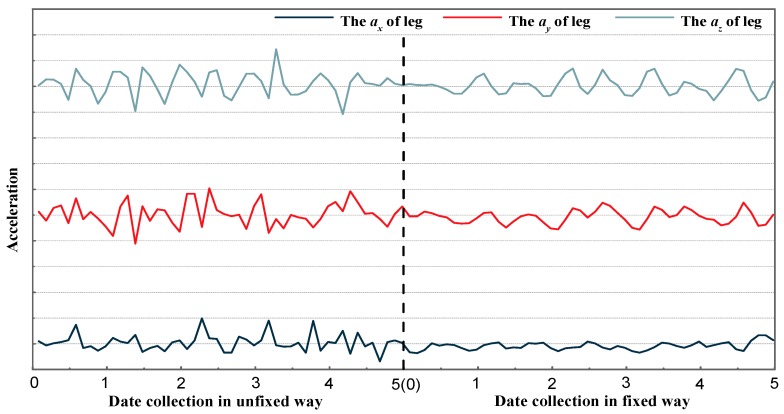
Sample acceleration data of walking based on smartphones based on two ways.

Since two smartphones are used to acquire data, there is an acquisition time difference between two devices. Moreover, the time difference will be accumulated to a greater extent in a long period of acquisition time, which will affect the accuracy of activity recognition. In such cases, utilizing a preprocessing technique to control the time difference and select the appropriate time interval can reduce related errors. Since the frequency of data acquisition was set at 10 Hz, i.e., the time interval for collecting data was 0.1 s, a criterion was established in which the time interval between two smartphones was within 0.1 s to filter unrelated sets of data. Figure 4 shows the data signals obtained by unfixed and fixed methods. It can be seen that the fixed data-acquisition method will reduce the influence of noise. Hence, the smoothing of data can be ignored.

### 3.3. Feature Extraction

Features represent specific properties of the initial data. From this perspective, they can be viewed as the description and characterization of the identified object. The data collected by smartphones was a set of discrete points of acceleration and angle information. Each item of three-axis acceleration or angle data only represented the acceleration state or incline angle of a certain part of a human body at a certain moment. However, these two states might appear in different activities at the same time, implying that one single data point could not describe or represent one unique activity. Hence, features of the initial data had to be extracted for further classification and identification.

David [18] summarized the commonly used features of sensor data and concluded that they are mainly in the time, frequency, and discrete domains. In subsequent studies, researchers have updated and improved the feature extraction of sensing data. Table 1 summarizes the commonly used features extracted from sensing data. This research only selected time domain features in order to reduce calculation time and simplify the calculation. The time domain, also known as signal statistical features, uses statistics to extract features. Since the calculation of the time domain is relatively simple, it is often adopted in the research of behavior recognition, particularly in fields with many requests in real time [11]. Common features of the time domain include mean, median, variance, standard deviation, covariance, max/min, interquartile range (IQR), zero-crossings rate (ZCR), skewness, kurtosis, root mean square (RMS), and signal vector magnitude (SVM). The mean, median, minimum, maximum, and standard deviation were extracted as features to identify seven actions such as walking, sitting, going up stairs, and driving [25]. In addition to the mean and variance, ZCR and RMS were extracted [15]. Similarly, IQR was extracted [6], and SVM was extracted from acceleration signals [26]. Hence, five common time domain features, namely, mean, standard deviation, IQR, skewness and covariance, used for activity classification and identification, were selected in this study and extracted from raw data as feature vectors. The mean, standard deviation, IQR, skewness, and covariance were calculated as shown in Equations (1)–(7).

1. Mean

The mean is usually used to smooth the entire dataset by eliminating peak points and noise of raw data. The formula is:

(1) $$\mathrm{mean}=\frac{1}{n}\sum_{i=1}^{n}s_{i},$$

where n is the number of data points and $s_{i}=\;\left[a_{x_{i}}^{1},a_{y_{i}}^{1},\;a_{z_{i}}^{1},\;g_{x_{i}}^{1},\;g_{y_{i}}^{1},\;g_{z_{i}}^{1},\;a_{x_{i}}^{2},a_{y_{i}}^{2},\;a_{z_{i}}^{2},\;g_{x_{i}}^{2},\;g_{y_{i}}^{2},\;g_{z_{i}}^{2}\right]$, $a_{x}^{1}$, $a_{y}^{1}$, and $a_{z}^{1}$ represent the three axis acceleration of the right wrist, $g_{x}^{1}$, $g_{y}^{1}$, and $g_{z}^{1}$ represent the three axis angel of the right wrist, $a_{x}^{2}$, $a_{y}^{2}$, and $a_{z}^{2}$ represent the three axis acceleration of the right leg, $g_{x}^{2}$, $g_{y}^{2}$, and $g_{z}^{2}$ represent the three-axis angle of the right leg.

2. Standard Deviation

The standard deviation is usually utilized to represent the degree of dispersion of the entire dataset and eliminate individual variability. The formula is:

(2) $$\mathrm{std}=\sqrt{\frac{1}{n-1}\sum_{i=1}^{n}{\left(s_{i}-\mathrm{mean}\right)}^{2}}.$$

3. Interquartile Range (IQR)

The interquartile range, also known as the inner distance, is the difference between the upper and lower quartiles. The IQR is used to measure the discreteness of a dataset. Since the interquartile range is mainly affected by data values in intermediate positions, it has the effect of eliminating noise. The IQR is calculated as follows:

The raw data $s_{i}$ is arrayed in ascending order to obtain $b_{i}$. The position of the quartile is shown in Equation (3), where j is the number of quantiles, $k_{j}$ is the integer part of $P_{j}$, and $r_{j}$ is the fractional part:

(3) $$P_{j}=1+\frac{\left(n-1\right)j}{4}.$$

The quartile is calculated as shown in Equation (4), where $Q_{1}$ is the upper quartile and $Q_{3}$ is the lower quartile:

(4) $$Q_{j}=b_{k_{j}}+\left(b_{k_{j}+1}-b_{k_{j}}\right)r_{j}.$$

Then the interquartile range is calculated as:

(5) $$\mathrm{IQR}=Q_{3}-Q_{1}.$$

4. Skewness

Skewness measures the degree of asymmetry of a probability density distribution curve compared to the average. The formula is:

(6) $$\mathrm{skewness}=\frac{\frac{1}{n}\sum_{i=1}^{n}{\left(x_{i}-\mathrm{mean}\right)}^{3}}{{\mathrm{std}}^{3}}.$$

5. Covariance

Covariance measures the degree of correlation between different axes in a coordinate system. The formula is:

(7) $$\rho_{x,y}=\frac{\sum_{i=1}^{n}(x_{i}-{\mathrm{mean}}_{x})\left(x_{i}-{\mathrm{mean}}_{y}\right)}{\sqrt{\sum_{i=1}^{n}{\left(x_{i}-{\mathrm{mean}}_{x}\right)}^{2}*\sum_{i=1}^{n}{\left(y_{i}-{\mathrm{mean}}_{y}\right)}^{2}}}.$$

### 3.4. Data Labelling

Following data preparation and feature extraction, the construction activity class labels was assigned to each window with the help of the video data. This step is to ensure that the sensor data over a period of time can correctly represent actual construction activities. In addition, it serves as the ground truth for the learning algorithm [3].

### 3.5. Supervised Learning

Machine learning is often adopted to seek and gain valuable information from a mass of data. Traditional machine learning uses two common learning algorithms, which are supervised and unsupervised learning. The supervised learning algorithms commonly used in the study of human motion and behavior recognition include support vector machines (SVMs), artificial neural networks (ANNs), decision tree, naive Bayes, and K-nearest neighbor (KNN). These algorithms all achieved satisfactory results in the field of human activity recognition and show great potentiality in construction workers’ activities identification as shown in Table 2.

Since the decision tree algorithm has always been used for human activity-recognition studies [5,6,9,25,30] and it has the advantage of dealing with complex and non-linear problems, it was utilized in this paper to classify experimental data from construction activities. In general, the classification process of a decision tree is based on optimal attributes with three criteria: information gain, gain ratio, and Gini index. The ID3 algorithm is mostly used to separate discrete datasets. Through a recursive method, the dataset can be transformed into a decision tree. However, the dataset segmentation is too fast to directly process continuous features. The C4.5 and CART algorithms can be used to construct binary trees and deal with continuous data. In addition, the CART algorithm cannot only solve classification problems effectively, but can implement a regression with different error criteria [24]. As a CART algorithm based on the Gini index was adopted in this research for use with continuous variables, this paper utilized the CART decision tree algorithm to classify and identify different construction activities.

### 3.6. Classification Model Verification and Evaluation

Since it was not clear whether the identification results agreed with reality, the classification model could not be used directly for activity recognition. It was necessary to evaluate the model’s recognition accuracy and verify its effectiveness. The hold-out and cross-validation methods are commonly used to evaluate and validate a model’s performance. Cross-validation is used to measure the performance of the classification model. In this method, the sample dataset is divided into 10 mutually exclusive subsets of similar size. Nine subsets are utilized as a training set and the remainder are used as a test set to conduct training and test repeatedly ten times. To reduce the error caused by different sample sizes of subsets, the ten-fold cross is carried out 10 times with a random division ratio. The average value of 10 ten-fold cross results is regarded as the final evaluation result.

### 3.7. Activity Identification

In a complex construction environment, sample data of construction workers can be collected in advance to create a database that is subsequently imported into the classification model for training. In the course of activity classification, the new collected datasets are viewed as test sets for classification and identification, realizing the intelligent management and supervision of workers’ on-site activities. After the tests for new datasets are completed, they are added in turn to the sample database as a training set for the next test. Based on this, the constantly expanding training set can enhance the performance of the training model by improving the precision of its predictions.

## 4. Experiment Setup

In this research, experiments were carried out on an outdoor workspace where activities performed by subjects were imitated. Armbands applied in sports were used to fix smartphones on the right wrist and upper right leg of each subject. The study on the selection of accelerometer positions for activity recognition has indicated that the lower left arm and the upper right arm are the two best positions to yield the highest information gain [17]. Considering the practicalities of workers’ daily activities, the lower left arm was not selected in the study. Instead, the selection of right wrist and upper right leg were expected to provide accurate and consistent signals compared to other positions of human body. In view of the differences among various subjects, nine subjects (five males and four females) with different heights and weights participated in the experiment. Each subject was an engineering management student who had basic construction engineering knowledge and experience in working at construction sites. The data collection for each experiment was repeated five times. During the experiment, an experiment facilitator recorded the process of each experiment for a further data filter and annotation. Moreover, subjects in the experiments were not guided to perform activities in a specific way, they accomplished their own work in their natural poses and states.

### 4.1. Data Acquisition

**Figure 5:**
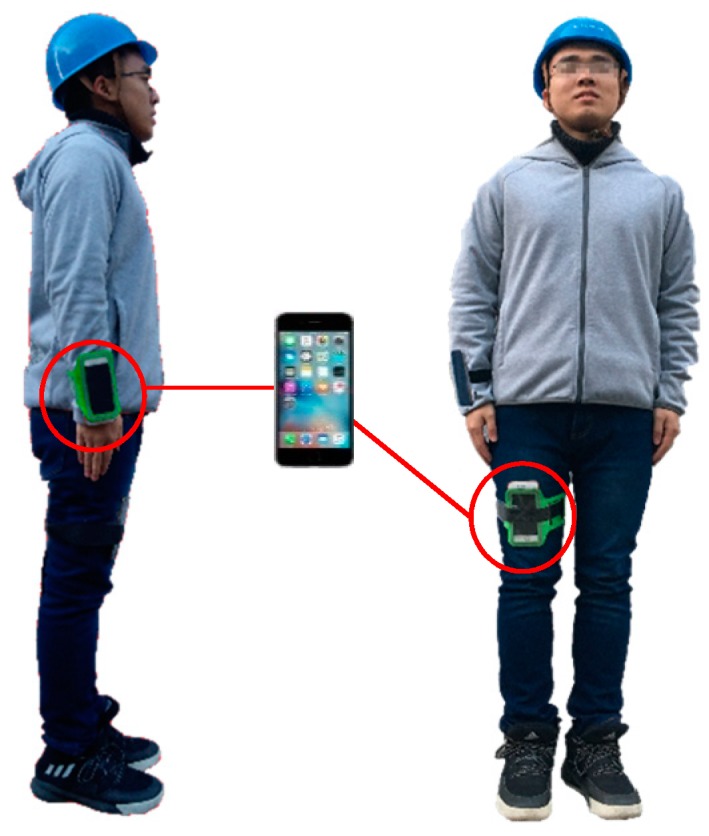
Schematic diagram of smartphone location.

**Figure 6:**
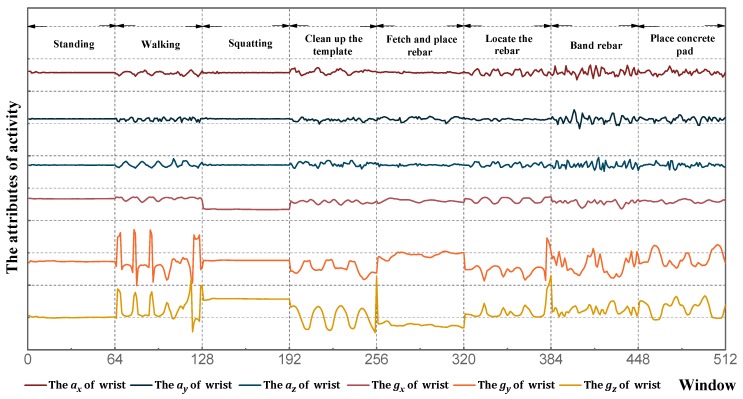
Initial waveform of wrist.

**Figure 7:**
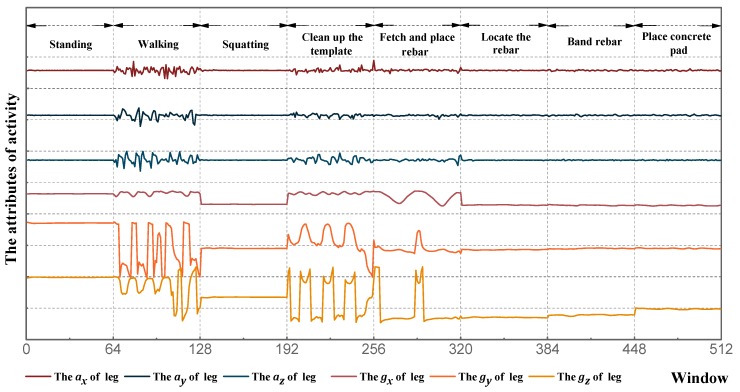
Initial waveform of leg.

Before collecting experimental data, smartphones were fixed on the subject’s right wrist and right leg to reduce noise data, as shown in Figure 5. The frequency and collection times of data acquisition were set as 10 Hz and 60 s, respectively. This sampling frequency has also been used in previous research for sensor-based activity identification [31,32]. At the end of data acquisition, Orion-CC was utilized to extract the experimental data, for a grand total of 360 sets. Figure 6 displays the initial waveform of eight activities of the wrist, and Figure 7 displays the initial waveform of eight activities of the leg. Each activity includes 12 dimensional attribute values: $a_{x}$, $a_{y}$, and $a_{z}$ of the wrist; $g_{x}$, $g_{y}$, and $g_{z}$ of the wrist; $a_{x}$, $a_{y}$, and $a_{z}$ of the leg; and $g_{x}$, $g_{y}$, and $g_{z}$ of the leg.

### 4.2. Feature Extraction

Before classification and identification, feature vectors must be extracted from raw data, providing a prerequisite for better performance in classification and identification [33].

**Figure 8:**
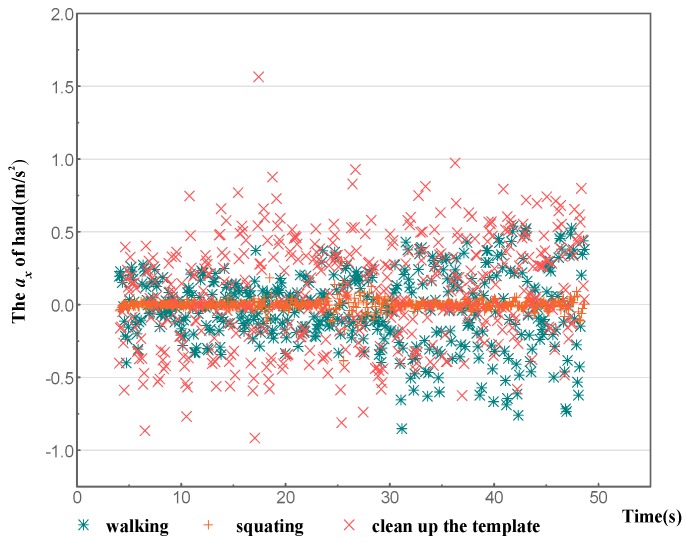
Scatter plot of ***a_x_*** for walking, squatting, and cleaning up the template.

**Figure 9:**
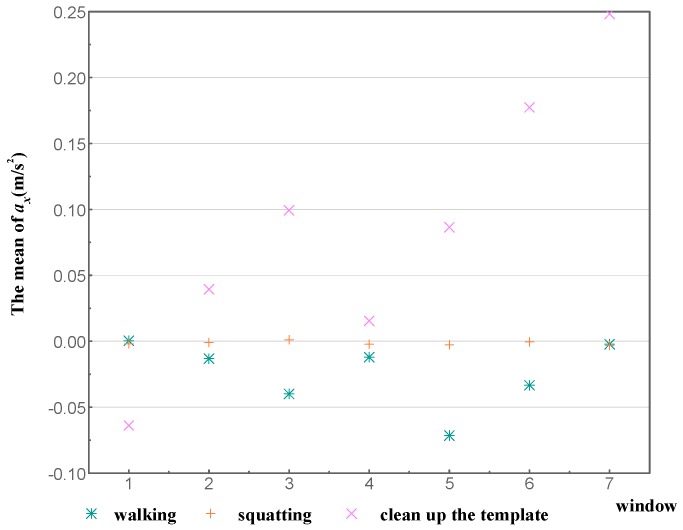
Scatter plot of mean for walking, squatting, and cleaning up the template.

An activity is the concentrate expression of various states in serial time. Therefore, the features of each activity were extracted from data over a certain time period. An appropriate time window was determined to segment initial data to implement feature extraction. Previous research on segment lengths for human activity identification shows that identification accuracy performed well with a segment length of 6.4 s [34] In addition, studies for sensor-based activity identification have suggested a 50% overlap between the adjacent windows [35,36,37]. Therefore, to guarantee the accuracy of classification and identification, the time window of feature extraction was set to 6.4 s and the repetition rate was 50%. After feature extraction, the dimensional features of each activity increased from 12 to 60, making the characteristic features more apparent. Figure 8 shows the scatter plot of raw X-axis acceleration from the wrist versus time. The three activities of walking, squatting, and cleaning up the template were selected. It can be seen that the data points of these three activities overlap often with obscure characteristic features. It is particularly difficult to separate walking and cleaning up the template. Figure 9 presents the scatter plot of mean X-axis acceleration from the wrist versus time, with the same time period as in Figure 8. The characteristic features of X-axis acceleration in each activity are obvious, and the division boundaries are clear.

### 4.3. Activity Classification and Recognition

Although the collected data are discrete in time, the properties of attributes can be regarded as continuous. Hence, a CART algorithm based on the Gini index was adopted in this research for use with continuous variables. The algorithm was compiled and realized in Python 3.5 with Notepad ++ as the programming platform. While constructing the decision tree, the tree structure was stored through a data structure dictionary embedded in Python. The pre-pruning error-correction factor was set as 0.5, and the over-fitting of the decision tree was controlled by adjusting the parameters. After generating the decision tree model by the specific recursive function create Tree, a cross-validation method was used to validate and evaluate the model. We selected ten-fold cross-validation, and the average values were used as assessment results.

A decision tree algorithm can be utilized to identify construction workers’ activities after its effectiveness is validated. As for the selection of recognition data, 20% of the sample set was randomly extracted as a testing set. An evaluation system based on precision, recall, F1-score, and a confusion matrix was subsequently built.

## 5. Results and Discussion

In the stage of classification training and identification, the individual samples of each subject and the overall samples were classified and predicted. The classification and prediction results of individual samples are shown in Table 3. In the test of individual sample classification, the ratios for training and testing were set to 0.9 and 0.1, respectively, and the average accuracy of ten-fold cross-validation was used as the final result of classification. In addition, 20% of the samples were selected to implement the prediction.

Data analysis indicated that due to the differences of each individual sample, the accuracy of activity classification and prediction failed to reach 100%. Moreover, the necessity of collecting individual samples repeatedly during data acquisition was proved. Because of the differences between individuals, the accuracy of classification and prediction of each individual were also different. Therefore, it was necessary to collect data from multiple workers to build the training set. According to Table 3, the average accuracy of classification for individual samples based on the CART algorithm can reach 95.45%, and the average accuracy of prediction is as much as 92.98%. Further analysis showed that the accuracy of prediction was generally lower than that of classification.

To verify the universality of the decision tree algorithm, the classification and prediction of samples were implemented. The results are shown in Table 4. During classification, the ratios for training and testing were set to 0.9 and 0.1, respectively. The average accuracy of ten-fold cross-validation was used as the final result of classification. In addition, 20% of the individual samples were selected to implement prediction. Compared with individual samples, the classification accuracy of overall samples decreased by 5.6%, while the prediction accuracy increased by 1.93%, which pointed out that increasing the sample size of the training set and constantly improving the database will enhance the accuracy of prediction to some degree.

Table 5 shows the prediction result of each activity. The interval ranges of precision, recall, and F1-score were 0.76 to 1.00, 0.82 to 1.00, and 0.79 to 1.00, respectively. Specifically, the precision, recall, and F1-scores of standing, walking, squatting, cleaning up the template, fetching, and placing the rebar were close to 1.00, while the indicators of locating the rebar, binding the rebar, and placing the concrete pad were relatively lower. In particular, the recall and F1-score did not reach 0.9. The distinction between the first five activities and the others was apparently due to their unique attributes, which caused the prediction to be hardly affected by other motions. In contrast, the prediction accuracy of locating rebar, binding rebar, and placing concrete pad was not satisfactory, which indicated that the distinction between these three activities and others was blurred. The subtle differences among various activities cannot be utilized to implement complete activity separation, leading to poor prediction performance.

**Figure 10:**
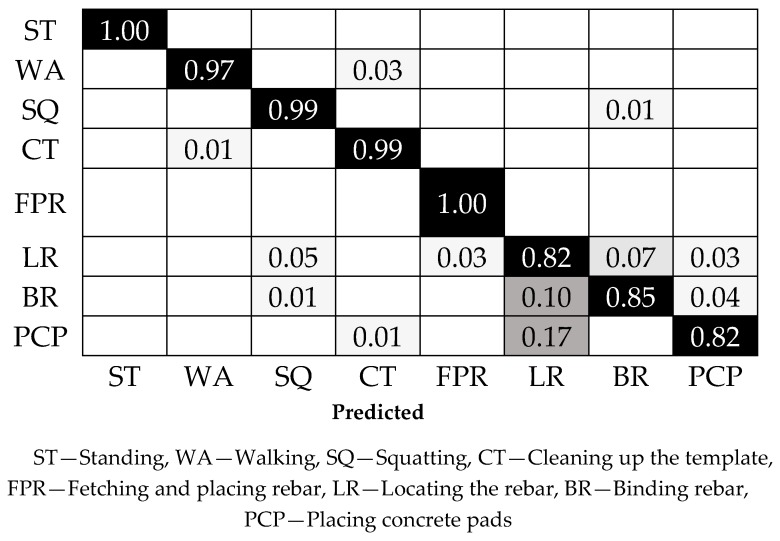
Confusion matrix of CART classification for eight activities.

The confusion matrix for activity classification is shown in Figure 10. The bottom coordinate of the confusion matrix represents the prediction results of activities, while the right coordinate represents the real labels of activities. Additionally, the diagonal data indicate the prediction accuracy of each activity and the remaining data stands for the false prediction rate. Further data analysis showed that the prediction accuracy of locating the rebar was only 82%, i.e., the remaining data points were wrongly predicted as squatting, fetching and placing the rebar, binding the rebar, and placing concrete pad. The wrong prediction of each activity was distributed uniformly. However, 17% of the data of placing the concrete pad and 10% of the data of binding the rebar were wrongly predicted as locating the rebar, which indicated that three activities, i.e., locating the rebar, binding the rebar, and placing the concrete pad, have a certain degree of similarity. Thus, it was hard to acquire satisfactory classification results based only on acceleration and angle information from the right wrist and right leg. In other words, the subtle differences in various activities could not be well measured and described using the above method.

## 6. Conclusions

Construction-process management has become a hot issue in both the construction industry and academia. Process management is currently implemented by positioning workers, material, and equipment to control cost, quality, progress, and safety. However, the research in management and surveillance of construction workers is relatively extensive and usually loses sight of details. Moreover, in the study of the activity-recognition of construction workers, only a few types of pre-set activities are selected, which provides limited reference values to construction managers. This paper proposes a method to identify the activity of construction workers based on three-axis acceleration and angle data collected through smartphones. The CART algorithm of a decision tree was adopted to classify eight activities. The average classification accuracy of individual samples reached 95.45%, while the classification accuracy of overall samples was 89.85%. In addition, the prediction accuracy of individual and overall samples was over 90%. During the experiment, the influence of noise was reduced by fixing data-acquisition tools, i.e., smartphones and time-series analysis. The five statistical features of mean, standard deviation, skewness, IQR, and covariance were utilized to improve the classification accuracy. The experimental results showed that the eight construction activities of standing, walking, squatting, cleaning up the template, fetching and placing the rebar, locating the rebar, binding the rebar, and placing the concrete pad could be classified and distinguished on the basis of three-axis acceleration and angle information collected from human wrists and legs. Additionally, the feasibility of using sensors embedded in smartphones to collect information generated by construction management was verified. Compared with traditional data-collection methods using external sensors, smartphones have remarkable advantages of convenience and efficiency. Over 90% recognition accuracy indicated that the integration of a decision tree algorithm and smartphones could be used to classify the complex activities of construction workers.

## 7. Future Work

Although the activity-recognition framework built in this study could achieve satisfactory accuracy in classification and prediction, it was only an experimental inquiry that needed improvement in both theory and practice. For instance, considering the special nature of construction workers, the data-acquisition tools should be attached in an unfixed way so as to decrease the impact on workers’ daily activities. The non-stationary data generated by an unfixed data-acquisition method can be de-noised and smoothed through a wavelet transform or the addition of windows. Additionally, in the applications of classification algorithms, multiple algorithms such as ANNs, SVMs, naive Bayes, and other classifiers should be combined to improve classification and prediction accuracy. In a complex construction environment, workers engaged in floor-reinforcing steel work use many kinds of motion. Thus, there is great development potential in the design and selection of workers’ activities.

A smartphone has the advantages of integrating multiple sensors to collect real-time information and powerful capabilities of data transmission, storage, and processing. The integrated performance gives it potential as an interactive platform for intelligent management in complex construction sites. A complete worker-activity recognition system can be constructed based on a smartphone’s hardware and software, including the built-in CPU and GPU processors, storage cards, operating system, and wireless adapters, to raise the level of intelligent management in the construction industry.

**Figure 11:**
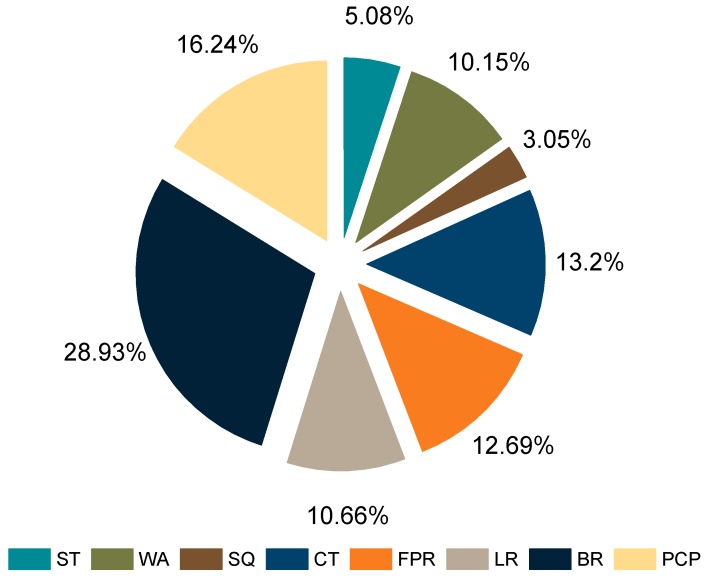
Time ratio of construction workers’ activities.

With the development of construction industrialization, the improvement of construction efficiency has attracted increasing attention from building enterprises. However, the scope of current research is limited to fields with special environmental characteristics, and these are not universally applicable. Moreover, as for influencing factors of construction efficiency, the angle of systematic analysis has an evident trend of polarization. Specifically, the analysis is too vague and macroscopic in light of issues of national policy, government support, enterprise-management systems, and employee education. In contrast, the analysis of the environmental impacts of certain regions and the limitations of special techniques is too one-sided. Therefore, based on the intelligent identification of construction workers’ activities, as shown in Figure 11, the operation time of each activity can be calculated to quantitatively evaluate the efficiency of workers. Moreover, it will help managers to identify the critical factors that affect construction efficiency and assist managers in making correct decisions and judgments.

**Figure 12:**
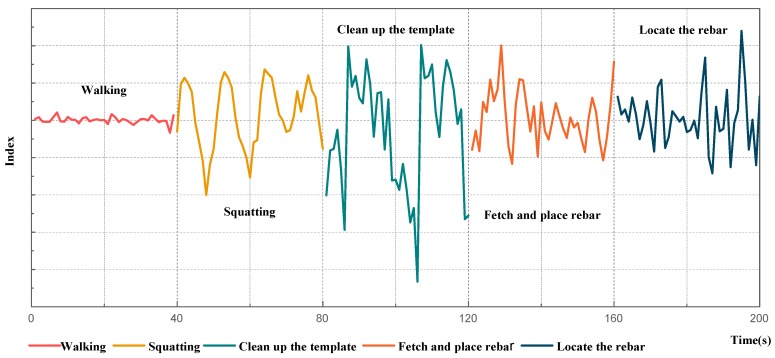
Visualization of construction process.

Floor-reinforcing steel work has a unique construction technology and construction process. Based on intelligent recognition of workers’ activities, daily construction activities in a certain period of time can be visualized, as shown in Figure 12. Managers can judge whether there is an absent or duplicated construction procedure according to the map of the construction process. Combined with field conditions in construction sites, a better solution to existing problems can be proposed. For instance, if there is too much auxiliary work in the construction process, managers can reduce unnecessary work by optimizing the construction work platform. Additionally, working procedures can be adopted to regulate workers’ construction activities.

## Figures and Tables

**Table 1:** Summary of features.

Time Domain	Frequency Domain	Discrete Domain
Mean, Median Variance, Standard deviation Max, Min, Range Interquartile range (IQR) Skewness Kurtosis Root mean square (RMS) Integration Correlation, Cross-correlation Zero-crossings Signal magnitude area (SMA) Signal vector magnitude (SVM)	Coefficients sum	Euclidean-based distances
	DC component	Levenshtein Edit distance
	Spectral entropy	DTW distance
	Information entropy	
	Spectral analysis of key coefficients	
	Frequency-domain entropy	

**Table 2:** Human activity recognition based on different supervised learning algorithms.

Classifiers	Human Activity	Recognition Accuracy (%)
Decision tree	Running, Walking, Sitting	92.64% [6]
	Standing, Walking, Jogging, Upstairs, Downstairs	92.30% [27]
Naive Bayes	Cycling, Vehicle, Running, Stationary, Walking	93.87% [7]
SVM	Walking Treadmill, Running, Running Treadmill, Going Upstairs, Going Downstairs, etc.	92.40% [12]
K-nearest neighbor (KNN)	Lying, Sitting, Standing, Walking, Running, Jumping	78.23% [28]
Artificial neural networks (ANN)	Walk, Slip, Trip	94.00% [29]

**Table 3:** Classification and prediction accuracy for individual samples.

Subject	Classification Accuracy (%)	Prediction Accuracy (%)
Subject 1	97.78%	90.12%
Subject 2	94.27%	90.02%
Subject 3	92.05%	88.07%
Subject 4	97.55%	98.76%
Subject 5	95.11%	92.59%
Subject 6	97.11%	93.82%
Subject 7	92.11%	88.89%
Subject 8	95.12%	97.53%
Subject 9	97.94%	97.04%
Average	95.45%	92.98%

**Table 4:** Classification and prediction accuracy for collective samples.

Index	Precision	Recall	F1-Score	Classification Accuracy	Prediction Accuracy
Result	94.91%	94.91%	94.91%	89.85%	94.91%

**Table 5:** Result of eight activities’ prediction.

Activities	Precision	Recall	F1-Score
Standing	1.00	1.00	1.00
Walking	0.98	0.97	0.97
Squatting	0.93	0.99	0.96
Cleaning up the template	0.96	0.99	0.97
Fetching and placing rebar	0.98	1.00	0.99
Locating the rebar	0.76	0.82	0.79
Binding rebar	0.91	0.85	0.88
Placing concrete pads	0.92	0.82	0.87
